# Severe dengue encephalitis showcasing the “double doughnut sign” on MRI: A case report

**DOI:** 10.1016/j.radcr.2025.06.043

**Published:** 2025-07-05

**Authors:** Salhadin Mohammed, Aman Edao Bime, China Tolessa Sedi, Hussen Nuri Saliha, Surafel Tilahun Maru, Abdulfatah Workicho Mustafa

**Affiliations:** a Internal Medicine Department, Division of Neurology, School of Medicine, College of Health Sciences, Wollo University, Dessie, Ethiopia; b Anaesthesiology, Critical Care, and Pain Medicine Department, School of Medicine, College of Health Sciences, Haramaya University, Harar, Ethiopia; c Neurosurgery Department, School of Medicine, College of Health Sciences, Addis Ababa University, Addis Ababa, Ethiopia; d Neurology Department, School of Medicine, College of Health Sciences, Addis Ababa University, Addis Ababa, Ethiopia; e Radiology Department, School of Medicine, College of Health Sciences, University of Gondar Comprehensive Specialized Hospital, Gondar, Ethiopia

**Keywords:** Severe dengue, Dengue encephalitis, Double doughnut sign, MRI, Case report

## Abstract

Severe dengue can lead to fatal complications, including dengue encephalitis. We report a 14-year-old girl with severe dengue who presented with bilateral thalamic hemorrhagic infarction, indicated by distinct brain imaging findings (the so-called “double doughnut sign”), which is a rare presentation of a common infection. Positive dengue antigen and IgM tests were noted. The patient required critical care, including intubation and resuscitation, but was discharged improved and continues follow-up at our neurology clinic. This case highlights that dengue infection can be complicated by meningoencephalitis, and a high index of suspicion is needed to diagnose our patients promptly. Furthermore, imaging modalities such as MRI play a key role in diagnosing and managing patients with dengue encephalitis.

## Introduction

Dengue is an acute febrile illness caused by infection with 1 of 4 dengue viruses (DENV) belonging to the genus *Flavivirus*. Transmission occurs via the mosquitoes *Aedes aegypti* or *Aedes albopictus* during the process of taking a blood meal. *Aedes aegypti* and DENVs are endemic in every continent except Europe and Antarctica, although epidemic dengue hemorrhagic fever is more prominent in Asia and the Americas [1]. With an estimated 390 million infections occurring each year worldwide and over 2.5 billion individuals at risk [2], the DENVs are significant arthropod-borne viruses from both medical and public health perspectives.

Virtually, there are 3 distinct phases of infection: the febrile phase, the critical phase, and the recovery phase. The critical phase is not present in all infections unless they are complicated or severe [1]. Severe dengue infection encompasses a spectrum of manifestations, including at least one of the following: severe plasma leakage, characterized by shock, fluid accumulation, and/or respiratory distress; severe bleeding diathesis; severe organ involvement (liver, kidneys, or the brain) [1]. The incidence of severe dengue is rare, with the likelihood of developing complications being highest among people who are infected a second time by a different DENV type (approximately 18 months later) from the first infection [3].

Neurological complications from severe dengue are quite rare, and involvement of either the peripheral (e.g., Guillain-Barré Syndrome [GBS]) or, more commonly, the central nervous system (CNS) is a possibility [4]. CNS complications may include encephalopathy, which can result from hepatic failure or metabolic disturbances, as well as meningoencephalitis due to direct viral invasion. Other manifestations can include seizures, acute strokes, acute disseminated encephalomyelitis (ADEM), or a combination of these issues [4].

In such situations, imaging techniques such as MRI are essential tools for assessing the severity of dengue infection and any related neurological problems. Neuroimaging may reveal signs of encephalitis and meningitis resulting from the virus's invasion of the nervous system. Additionally, other systemic complications, such as metabolic or seizure-related encephalopathy and hemorrhagic stroke due to low platelet counts (thrombocytopenia), can also be detected [5].

The double doughnut sign is a notable radiological finding that can appear on an MRI of viral encephalitis, particularly in cases of dengue encephalitis [6]. This sign has been reported in a limited number of patients [[7], [8], [9], [10], [11]] and refers to a specific pattern of signal changes in the bilateral thalami. On imaging, the double doughnut sign can be seen on T2/FLAIR, diffusion-weighted imaging, or susceptibility-weighted imaging (SWI). It manifests as bilateral round hyperintensities in the thalami, each with a low-signal core, creating the appearance of 2 doughnuts. This distinctive appearance is believed to result from bilateral thalamic hemorrhages occurring within surrounding edema and areas of diffusion restriction.

## Case presentation

### Patient information

A 14-year-old right-handed female student from Hargeisa, Somaliland, arrived at our emergency department presenting a concerning array of symptoms. Three days prior to her visit, she had been experiencing a high-grade fever accompanied by a severe, debilitating headache that affected her entire head, making even the slightest movement uncomfortable. In addition to these distressing symptoms, she reported widespread aches in her bones and muscles, resulting in significant fatigue and lethargy.

As her condition progressed, she began to notice unusual bruising and an increased tendency to bleed more easily than usual. Most alarming, however, was a noticeable decline in her level of consciousness, leading to confusion and unresponsiveness. Her past medical history was unremarkable, with no previous illnesses or chronic conditions reported, and there were no significant family medical histories that could suggest a hereditary issue. This situation raised immediate concern within the medical team as they worked to uncover the underlying cause of her troubling symptoms.

### Clinical findings

Her physical examination revealed an acutely sick-looking girl with a blood pressure of 70/50, pulse rate of 146 (weak), respiratory rate of 32, oxygen saturation of 85%, and a temperature of 39.4. Her general medical exam revealed conjunctival injection, bruising, and oozing at venipuncture sites, and diffuse coarse crackles on chest examination, while the tourniquet test was negative. The nervous system examination showed a comatose patient with a Glasgow coma scale (GCS) of 6/15 (E1V2M3). Pupils were small in size and reactive; corneal reflexes were present. On motor examination, she had comparable and symmetrical bulk, with no fasciculations. The power examination was difficult to assess, but there were no prominent lateralizing signs. The tone was spastic and muscle stretch reflexes were 3+ in the triceps, biceps, brachioradialis, patellar, and ankle areas. Plantar response was upgoing. Nuchal rigidity was observed; however, both Kernig's sign and Brudzinski's sign were negative.

## Diagnostic assessment

With this history and physical examination findings, a presumptive diagnosis of Septic shock with meningoencephalitis was entertained; workups to confirm the diagnosis and to rule out possible differential diagnoses soon followed. Findings on the basic and metabolic work-ups revealed lymphopenia (ALC of 190 cells/microL; normal values: 1000-4000 cells/microL), thrombocytopenia (platelet count of 38,000; normal values: 150,000-400,000), and elevated liver enzymes (AST, ALT, and ALP were elevated 6, 7, and 2.2 times the upper limit of normal respectively), with deranged coagulation profile (INR of 3.1; normally 0.8-1.2 in individuals not taking anticoagulant medications) (see details in Table 1). Testing for malaria came back negative.

**Table 1:** Baseline laboratory investigation summary of patient BM (patient’s initials), done on October 21, 2024.

Date: 21/10/2024	Laboratory investigations	Results	Laboratory normal reference range
	WBC	2700	3600-10,200
	Neutrophil %	88.4	43.5-73.5
	Lymphocyte %	7.1	15.2-43.3
	Hemoglobin	14.1 g/dL	12.5-16.3 g/dL
	Platelets	38,000	152,000-348,000
	Blood Film	Negative	Negative
	Urea	17 mg/dL	7-18 mg/dL
	Creatinine	0.6 mg/dL	0.6-1.3 mg/dL
	AST	240 units/L	<40 units/L
	ALT	280 units/L	<40 units/L
	ALP	400 units/L	<180 units/L
	Albumin	4 g/dL	3.5-5.5 g/dL
	PICT and VDRL	Negative	Negative
	HBsag and HCVab	Negative	Negative
	Dengue IgM and IgG	Positive	Negative
	Dengue antigen	Positive	Negative
		CSF analysis	
	Appearance	Clear	Clear
	WBC	300	0-5 WBCs/MicroL
	Neutrophil %	28.6	-
	Lymphocyte %	70.2	-
	Protein	56 mg/dL	<45 mg/dL
	Glucose	57 mg/dL	45-80 mg/dL
	Gram stain and culture	Negative	Negative
	Gene Xpert and AFB	Negative	Negative
	Coagulation profile		
	INR	3.1	0.8-1.2
	aPTT	38	25-34 s

Abbreviations: AFB, acid fast bacillus; ALP, alkaline phosphatase; ALT, alanine transaminase; aPTT, activated partial thromboblastin time; AST, aspartate transaminase; CSF, cerebrospinal fluid; dL, deciliter; g, gram; HBsag, Hepatitis B virus surface antigen; HCVab, Hepatitis C virus antibody; INR, International Normalized Ratio; mg, milligram; ml, milliliter; pg, picogram; PICT, provider initiated counselling and testing for HIV; VDRL, Venereal Disease Research Laboratory; WBC, white blood cell count; µIU, micro international unit.

**Fig. 1:**
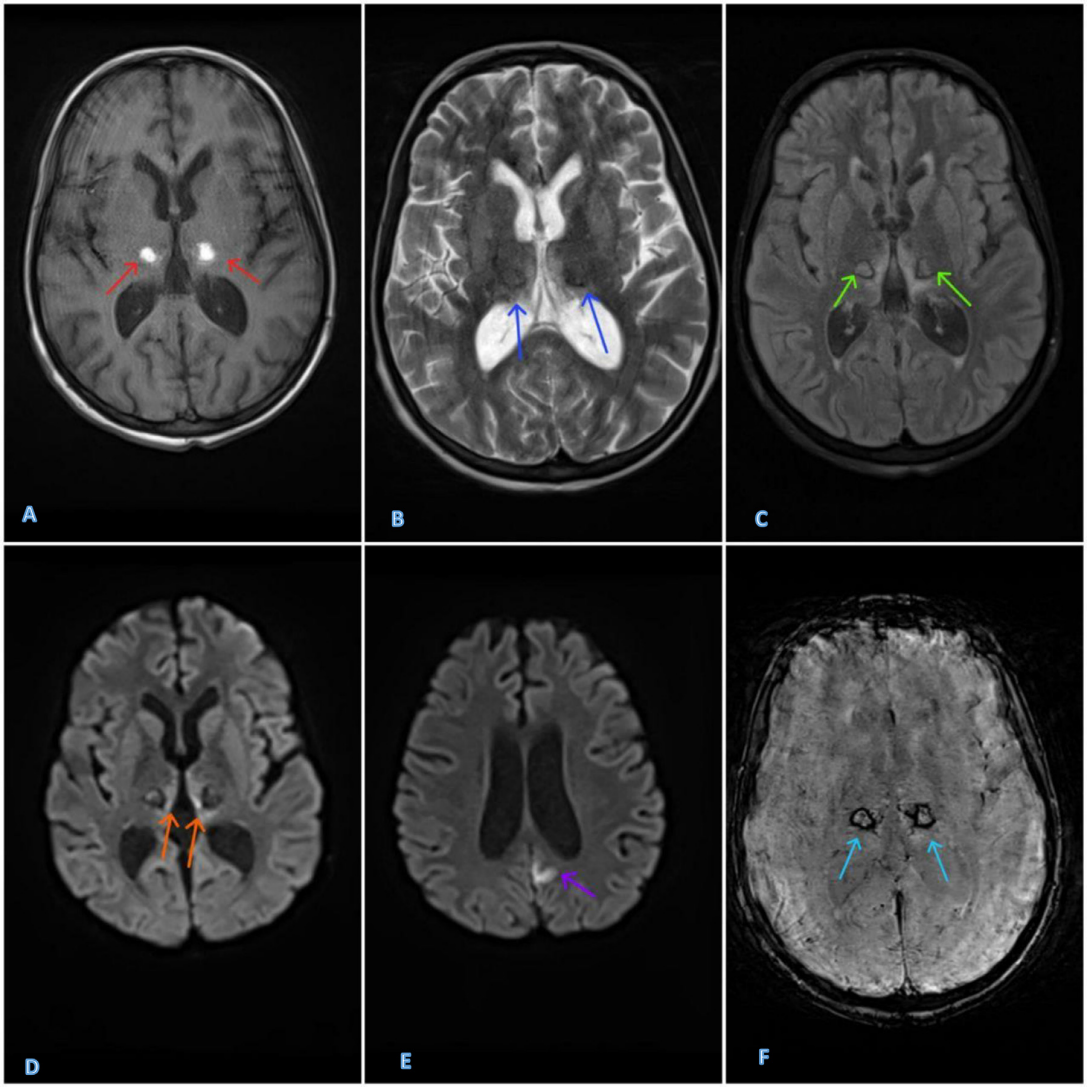
Brain MRI of a 14-year-old female patient showcasing the double doughnut sign, with evolution. There is a well-defined lesion in each thalamus, having T1 hyperintensity (A, red arrows), T2 hypo to isointensity (B, dark blue arrows), with fluid attenuated inversion recovery (FLAIR) and susceptibility weighted imaging (SWI) isointensity with a rim of hypointensity (C, green arrows and F, light blue arrows respectively). The paramedian parts of both thalami have restricted diffusion (D, orange arrows). There is also a small focus of cortical swelling and diffusion restriction in the parasagittal part of the left parietal lobe (E, purple arrow).

Further testing for dengue infection, which is endemic in Somaliland, confirmed the presence of both positive IgM antibodies and dengue antigen. Imaging conducted with a brain MRI showed bilateral thalamic hemorrhagic infarction (see Fig. 1). The cerebrospinal fluid (CSF) analysis indicated an aseptic lymphocytosis, with normal glucose levels and slightly elevated protein (see details in Table 1).

Considering our patient’s clinical presentation and investigation findings, a final diagnosis of severe dengue, complicated by dengue hemorrhagic fever, dengue shock syndrome, and dengue encephalitis was made.

## Therapeutic interventions

After the initial diagnosis was established, the patient was promptly transferred to the medical Intensive Care Unit (ICU) for intensive monitoring and management. Upon arrival, immediate airway management was prioritized due to the severity of the patient's condition. Mechanical ventilation was initiated to ensure adequate oxygenation and ventilation, allowing for optimal respiratory support.

Resuscitative measures were promptly implemented, which included administering intravenous (IV) fluids to restore hemodynamic stability. The use of vasopressors was also initiated to counteract hypotension, facilitating adequate perfusion to vital organs. During the insertion of the nasogastric tube, a significant finding occurred when approximately 200 mL of fresh blood was aspirated from the stomach, indicating potential upper gastrointestinal bleeding and necessitating further investigation.

To address coagulopathy and support the patient’s hemostatic needs, the medical team administered several units of platelets and fresh frozen plasma (FFP) for transfusion. This was crucial in managing any underlying clotting disorders and preventing further hemorrhagic complications. In parallel, broad-spectrum antimicrobial agents were administered to combat potential infections, as the patient was at high risk for sepsis due to their critical condition.

Throughout the 4-week stay in the critical care unit, the patient received ongoing comprehensive care, including regular monitoring of vital signs, laboratory tests, and imaging studies to track progress. The interdisciplinary team of healthcare professionals, including intensivists, nurses, and pharmacists, collaborated closely to adjust treatment protocols as needed based on the patient’s response.

Gradually, the patient’s condition stabilized, leading to a significant improvement in their clinical status. By the end of the 4-week postoperative period, the patient was deemed stable enough to be transferred to the medical ward for continued recovery. There, they received further rehabilitation and support as they regained strength and functionality.

After a total of 6 weeks of hospital care, the patient was discharged in a much-improved state, demonstrating considerable recovery and stability. They were provided with a comprehensive discharge plan, including follow-up appointments and instructions for at-home care to ensure ongoing health and recovery.

## Follow-up and outcome

The patient continued her follow-up appointments at our hospital over the following months until she achieved optimal neurological recovery. She was treated for central pain syndrome, which was a result of thalamic involvement, with carbamazepine. Additionally, she remained committed to her rehabilitation and physiotherapy program.

## Discussion and conclusion

In this case report, we present a 14-year-old critically ill girl exhibiting signs of severe dengue infection, featuring a rare occurrence known as the “double doughnut sign.” Notably, our patient displayed distinct signal intensity on brain MRI, following an initial CT scan that revealed bilateral circumscribed hypodense lesions in the thalamus.

Previous reports of the “double doughnut sign” in patients with dengue encephalitis describe T2/FLAIR hyperintensities, T1 iso/hypointensity, and restricted diffusion on DWI [[7], [8], [9], [10]]. In contrast, our patient showed well-defined lesions in both thalami with T1 hyperintensity and T2 isointensity, accompanied by a rim of susceptibility artifact. Additionally, the paramedian regions of both thalami exhibited restricted diffusion, suggesting early subacute hemorrhage with an ischemic base. The observed differences are related to the aging process of a hemorrhage that can be readily detected through MRI imaging.

In addition to the thalami, dengue encephalitis also typically involves the basal ganglia, cortical gray matter, and both subcortical and deep white matter [6]. In rare cases, lesions may appear in atypical locations such as the brainstem (especially the substantia nigra), cerebellum, and hippocampus [12]. In a case series involving 8 patients, MRI changes were observed in the supratentorial region (which includes deep periventricular white matter, subcortical white matter, and deep gray matter such as the basal ganglia and thalami), the infratentorial region (including the cerebellar white matter and brainstem, particularly the pons), and occasionally in the cortical gray matter. The MRI revealed mild to moderate hyperintensities on T2-weighted images and fluid-attenuated inversion recovery (FLAIR) sequences, with diffusion restriction evident on diffusion-weighted images [13].

Possible differential diagnoses for dengue encephalitis, which presents with distinct MRI findings, include acute necrotizing encephalopathy of childhood (ANEC), characterized by the presence of the trilaminar sign, and infections caused by other flaviviruses that can lead to encephalitis, such as Japanese encephalitis [6,14]. In tropical regions, it is essential to effectively rule out treatable conditions, including malaria, before proceeding with interventions for dengue encephalitis.

In conclusion, our case emphasizes that dengue infection can rarely complicate with meningoencephalitis, making it essential to maintain a high level of suspicion for timely diagnosis. Additionally, imaging techniques such as MRI are vital for guiding the diagnosis and management of patients with dengue encephalitis, adjunct to a thorough clinical assessment and evaluation.

## Patient consent

All methods were carried out in accordance with relevant guidelines and regulations. The patient’s data were collected after informed, voluntary, written, and signed consent was obtained from the hospital administrators and the study participant’s next of kin. To ensure the patient’s confidentiality, the name or personal identifier was not included, and all the data collected was kept confidential.
